# Supplementary material for: New Insights into the Behavior of NHC-Gold Complexes in Cancer Cells
Source: Pharmaceutics. 2023 Jan 31;15(2):466. doi: 10.3390/pharmaceutics15020466 (PMC9963827; doi:10.3390/pharmaceutics15020466)
Supplement: Supplementary file 1 [file pharmaceutics-15-00466-s001.zip › pharmaceutics-2089391-supplementary.pdf]

pharmaceutics

MDPI

Supplemetary

New insights into the behavior of NHC-Gold complexes in cancer cells

Giuseppa Augello [1,*], Antonina Azzolina [1], Filomena Rossi [2], Filippo Prencipe [3], Giuseppe Felice Mangiatordi [3], Michele Saviano [4], Luisa Ronga [5], Melchiorre Cervello [1] and Diego Tesauro [2,*]

1 Institute for Biomedical Research and Innovation (IRIB) CNR, Via Ugo LaMalfa 153, 90146 Palermo, Italy
2 Department of Pharmacy and Interuniversity Research Centre on Bioactive Peptides (CIRPeB), University of Naples "Federico II", via Montesano 49, 80131 Naples, Italy
3 Institute of Crystallography (IC) CNR, Via Amendola 122/O, 70126 Bari, Italy
4 Institute of Crystallography (IC) CNR, URT Caserta, Via Vivaldi 43, 81100 Caserta, Italy
5 E2S UPPA, CNRS, IPREM, Université de Pau et des Pays de l'Adour, 64000 Pau, France
* Correspondence: giuseppa.augello@irib.cnr.it (G.A.); dtesauro@unina.it (D.T.)

Keywords: NHC-gold(I) complexes; TrxR1 activity, antiproliferative effect, cancer cells

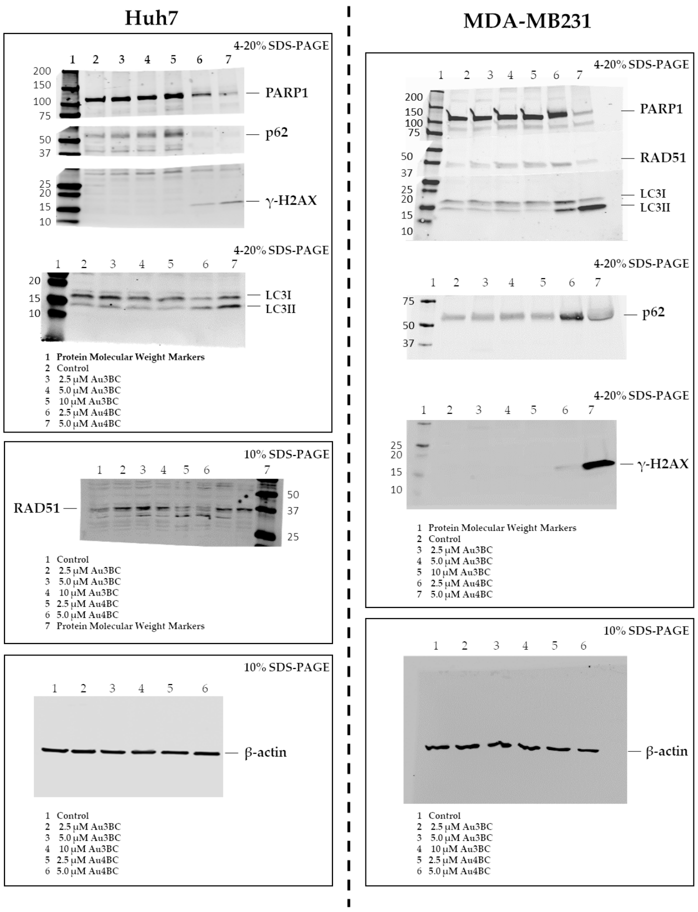

Figure S1. Original Western blot images.

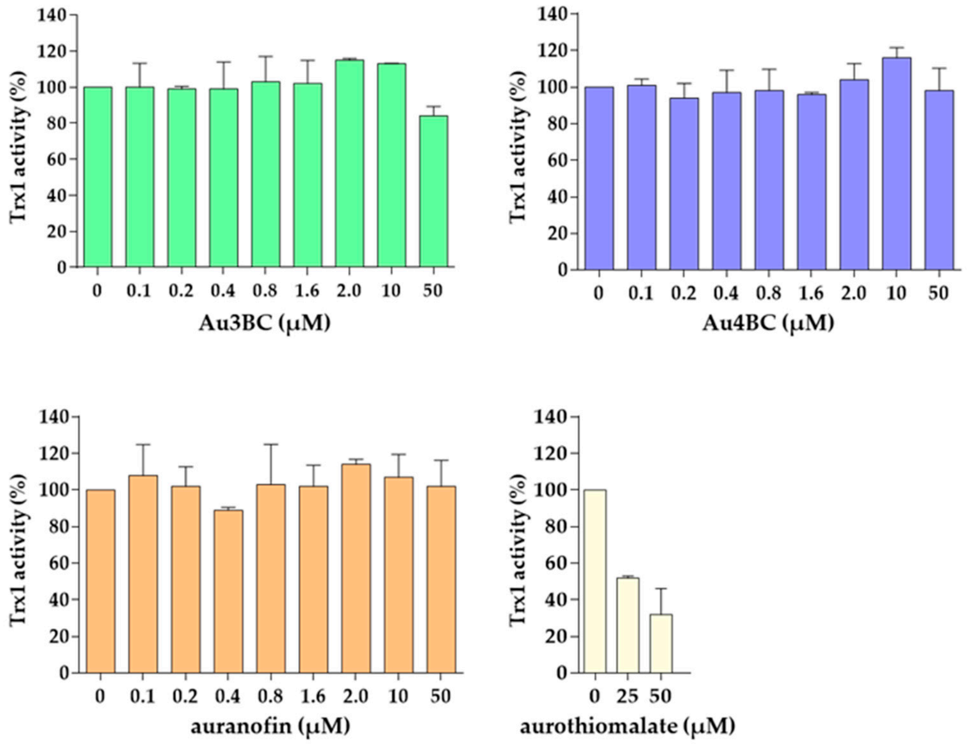

Figure S2. Trx1 activity in vitro. Human recombinant Trx1 was incubated with different concentrations of Au3BC, Au4BC and auranofin. Trx1 activity was assayed in presence of insulin and DTT at 25°C for 30 min. Aurothiomalate was used as Trx1 inhibitor positive control.
